# Combined Inhibition of the Renin-Angiotensin System and Neprilysin Positively Influences Complex Mitochondrial Adaptations in Progressive Experimental Heart Failure

**DOI:** 10.1371/journal.pone.0169743

**Published:** 2017-01-11

**Authors:** Laura Grois, Julian Hupf, Jörg Reinders, Josef Schröder, Alexander Dietl, Peter M. Schmid, Carsten Jungbauer, Markus Resch, Lars S. Maier, Andreas Luchner, Christoph Birner

**Affiliations:** 1 Department of Internal Medicine II, University Hospital Regensburg, Regensburg, Germany; 2 Institute of Functional Genomics, University Regensburg, Regensburg, Germany; 3 Electron Microscopy Core Facility, Institute for Pathology, University Hospital Regensburg, Regensburg, Germany; 4 Department of Internal Medicine I, Clinic St. Marien, Amberg, Germany; Virginia Commonwealth University, UNITED STATES

## Abstract

**Background:**

Inhibitors of the renin angiotensin system and neprilysin (RAS-/NEP-inhibitors) proved to be extraordinarily beneficial in systolic heart failure. Furthermore, compelling evidence exists that impaired mitochondrial pathways are causatively involved in progressive left ventricular (LV) dysfunction. Consequently, we aimed to assess whether RAS-/NEP-inhibition can attenuate mitochondrial adaptations in experimental heart failure (HF).

**Methods and Results:**

By progressive right ventricular pacing, distinct HF stages were induced in 15 rabbits, and 6 animals served as controls (CTRL). Six animals with manifest HF (CHF) were treated with the RAS-/NEP-inhibitor omapatrilat. Echocardiographic studies and invasive blood pressure measurements were undertaken during HF progression. Mitochondria were isolated from LV tissue, respectively, and further worked up for proteomic analysis using the SWATH technique. Enzymatic activities of citrate synthase and the electron transfer chain (ETC) complexes I, II, and IV were assessed. Ultrastructural analyses were performed by transmission electron microscopy. During progression to overt HF, intricate expression changes were mainly detected for proteins belonging to the tricarboxylic acid cycle, glucose and fat metabolism, and the ETC complexes, even though ETC complex I, II, or IV enzymatic activities were not significantly influenced. Treatment with a RAS-/NEP-inhibitor then reversed some maladaptive metabolic adaptations, positively influenced the decline of citrate synthase activity, and altered the composition of each respiratory chain complex, even though this was again not accompanied by altered ETC complex enzymatic activities. Finally, ultrastructural evidence pointed to a reduction of autophagolytic and degenerative processes with omapatrilat-treatment.

**Conclusions:**

This study describes complex adaptations of the mitochondrial proteome in experimental tachycardia-induced heart failure and shows that a combined RAS-/NEP-inhibition can beneficially influence mitochondrial key pathways.

## Introduction

Systolic heart failure is characterized by a detrimental activation of the sympathetic nervous system (SNS) and the renin-angiotensin system (RAS) [1–3], whose pharmacologic blockade has proven to be prognostically beneficial, respectively [4–7]. Nevertheless, facing a five-year survival rate of about 50% prognosis remains very poor [8] thereby indicating that the therapeutic potential has by far not been realized yet.

Having said this, increasing evidence points to a new pathophysiologic paradigm, where the true driving force for progressive left ventricular dysfunction is now seen in a deleterious imbalance between maladaptive (i.e., SNS and RAS) and protective (mainly the natriuretic peptide system, NPS) mechanisms [9], which means, that beneficial effects were to expect not only from inhibiting the former, but also from augmenting the later ones. Consequently, a new pharmacologic class has been developed which inhibits both the angiotensin converting enzyme and the natriuretic peptides degrading enzyme neprilysin [10]. The leading substance of this “vasopeptidase inhibitors” (VPIs) named class, omapatrilat, was thoroughly evaluated [11–13], but failed to be launched due to its rare, but relevant side effects (mainly angioedema). Subsequently, a neprilysin inhibitor was combined with an angiotensin-receptor blocker instead of an ACE-inhibitor, thereby introducing the class of ARNIs (angiotensin receptor neprilysin-inhibitors). Its leading substance, LCZ696, has recently shown beneficial effects with better tolerance and convincingly confirmed the new pathophysiological concept behind this combined RAS-/NEP-inhibition [14]. By further evaluating this principle, our group was able to demonstrate a positive impact of omapatrilat on structural cardiac remodeling and neurohumoral activation [15], which both could provide a pathophysiologic fundament for the beneficial clinical effects.

Besides this new paradigm of neurohumoral imbalance, a rapidly growing body of evidence points to a central role of mitochondrial impairment in progressive heart failure resulting in detrimental energetic deprivation and deleterious oxidative stress [16]. This was also confirmed by our work group when evaluating proteomic alterations in left ventricles [17] and atria [18]. But despite recognition of its importance, mitochondrial adaptations remain nevertheless insufficiently characterized during progression to overt heart failure and therefore deserve further evaluation to potentially identify new therapeutic targets. Furthermore, it is unknown which impact a combined RAS-/NEP-inhibition has on energetically relevant pathways and whether these two mechanisms are interlinked to result in beneficial clinical effects.

We therefore evaluated in our well established model of progressive, pacing-induced heart failure in rabbits [17,19,20,15,21,22], which structural, functional and proteomic alterations cardiac mitochondria undergo in different stages of heart failure, and whether these adaptations are influenced by combined RAS-/NEP-inhibition. Facing the evident importance of both neurohumoral and energetic mechanisms, we hypothesized that mitochondrial adaptations which develop in progressive heart failure should be reversed or at least mitigated by RAS-/NEP-inhibition.

## Methods

### Model of progressive pacing-induced heart failure

**Fig 1:**
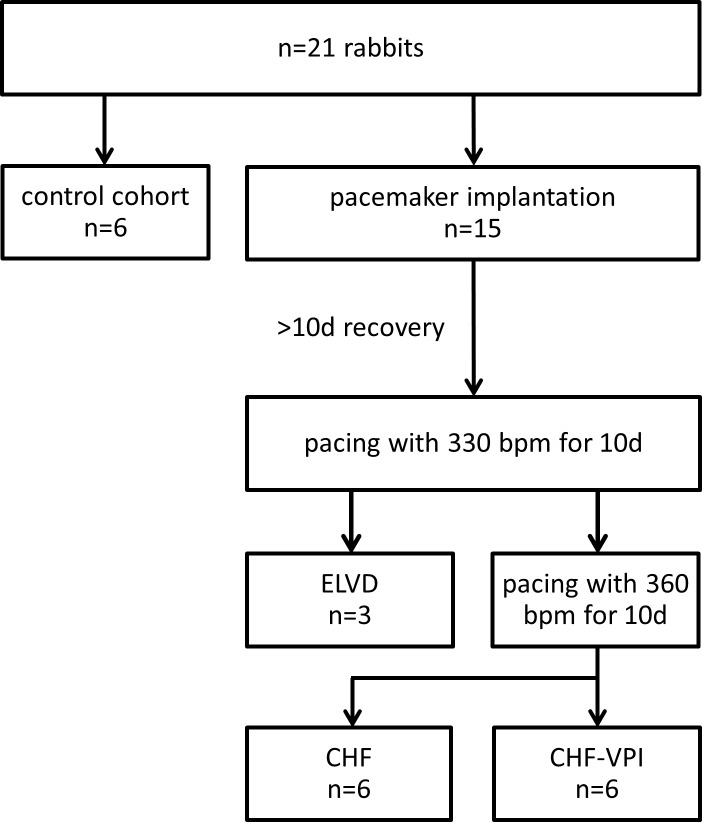
Study protocol.

All experiments were approved by the institutional and governmental animal care committees, respectively. A total of 21 male rabbits (chinchilla bastard; Charles River Laboratories International, Inc.) was used for this study (see Fig 1). The animals were exposed to a 12:12 h light:dark rhythm and received standard chow and water ad libitum. 15 animals underwent implantation of a cardiac pacemaker (Medtronic Minix 8340, Minneapolis, MN or Vitatron Model 810, Dieren, NL) and a transvenous right ventricular lead with 3 and 12 of them being paced until early left ventricular dysfunction (ELVD) and congestive heart failure (CHF) was generated, respectively. Surgical procedures were conducted under general anesthesia (Ketamine 60 mg/kg and Xylazine 5 mg/kg i.m.), and a standardized pharmacological protocol was applied in the early post-surgery period (4 mg/kg BW Rimadyl s.c. and 5 mg/kg BW Baytril s.c. for 3 days, respectively). Six untreated animals served as controls (CTRL). ELVD and CHF was induced by a standardized protocol of progressive rapid right ventricular pacing as described previously [17,19,20,15,18]. In brief, animals of the ELVD group were paced with 330 beats per minute (bpm) for 10 days, and animals of the CHF group underwent an additional pacing period at 360 bpm for further 10 days. Drinking water of CHF animals was either substituted with Omapatrilat to reach a daily dose of 50 mg/kg BW (CHF-VPI group), or remained untreated (CHF group). Pharmacological intervention was started after initiation of cardiac pacing and was sustained during the whole pacing period. At the end of the experiments, rabbits were euthanized by i.v. pentobarbital injection and tissue was rapidly harvested and deep-frozen. During the experiments, rabbits were monitored at least daily, which also included an evaluation regarding prespecified early endpoints (e.g., reduced food or liquid intake, dyspnea, impaired mobility, untreatable surgical complications such as severe infections). No early endpoint was met during the experiments and no animals died prior to the experimental endpoint. All experiments were approved by the institutional and governmental animal care committees, respectively (University Hospital Regensburg and Regierung der Oberpfalz).

### Echocardiography and hemodynamic evaluation

Measurements were done as described previously [15]: Under light sedation (5 mg midazolam i.m.) a long and short-axis echocardiogram (using HP Sonos 5500 with a 12 MHz probe) was performed in a supine position from the left parasternal window. LV enddiastolic (LVEDd) and LV endsystolic (LVESd) diameters, diastolic and systolic thickness of interventricular septum (IVSd, IVSs) and posterior wall (LVPWd, LVPWs) as well as left atrial diameters (LAd) were determined from three repeated 2D-guided M-mode tracings using the ASE conventions. Fractional shortening (FS) was calculated as: FS = (LVEDd-LVESd)/LVEDd.

Conscious arterial blood pressure was measured invasively via the medial ear artery under light sedation and after pausing the pacemaker stimulus.

Echocardiographic and hemodynamic evaluations were done at baseline, after each pacing period and at the end of the experiments.

### Isolation of cardiac mitochondria

A modified protocol by Schaeffer et al. [23] was used for mitochondrial isolation. Left ventricular tissue samples were first washed in 3 mL of isolation buffer (300 mM succrose, 10 mM HEPES, 0.2 mM EDTA, pH 7.4), than roughly chopped with a scalpel. Then, tissue samples were digested for 10 min in trypsin buffer (0.45 mg trypsin in 3 ml of isolation buffer). Afterwards, digestion was stopped by adding a trypsin inhibitor in 3 ml of isolation buffer with BSA added (1mg/ml), the supernatant was discharged and the tissue sample homogenized in 2ml of isolation buffer with BSA (1mg/ml) using a dounce homogenisator. After complete homogenization, the solution was centrifuged for 1 min at 600 g. The supernatant was again centrifuged for 15 min at 8000 g, resulting in a pellet with the isolated mitochondria. After discharging the supernatant, the pellet was resuspended in isolation buffer with BSA and again purified by centrifugation at 8000 g for 15 min. Finally, the pellet was resuspended in 100 to 150 μl of isolation buffer depending on its size. Protein content of isolated mitochondria was assessed by 2D Quant Kit (GE Healthcare) and photometry. Furthermore, efficiency of mitochondrial isolation was assessed by transmission electron microscopy and western blotting using antibodies against VDAC2, TOMM70, cytochrome c, IMMT and beta-actin (serving as control for cytosolic proteins).

### Protein expression of CPT1A (carnitine palmitoyltransferase 1 A)

The expression level of CPT1A, which is an essential enzyme for the beta oxidation of long chain fatty acids by mediating their transport from the cytosol into the mitochondrial intermembrane space, was determined by western blot analysis. For this purpose, anti-CPT1A (PA5-29995, Thermo Scientific, Waltham, USA) was used as primary antibody, and ab97085 Dnk pAb to rabbit-HRP (Abcam, Cambridge, UK) as secondary antibody.

### Enzymatic activites of citrate synthase, NADH dehydrogenase (complex I), succinate dehydrogenase (complex II), and cytochrome c oxidase (complex IV)

Activity of citrate synthase was assessed following the protocol of Srere et al. [24] In brief, mitochondrial membranes were destroyed by Triton X100, and acetyl-CoA, oxalacetate and DTNB were added. After reaction of oxalacetate with acetyl-CoA, which is mediated by citrate synthase (at 30°C), free CoA can convert DTNB into TNB. The amount of TNB can then be assessed by photometry at 412 nm over 200 s.

Activity of the ETC complex I-enzyme NADH dehydrogenase was determined as previously described [25]. In brief, this assay photometrically measures oxidation of NADH at a wavelength of 340 nm and additionally determines rotenone-insensitive complex I activity for control purposes. To assess enzymatic activity of the ETC complex II-enzyme succinate dehydrogenase, 20 μl of a mitochondrial isolate were mixed with warmed reaction medium containing 500mM phosphate buffer (pH 7.5), 50 mg/ml BSA, 200mM succinate, 5mM DCPIP, 10mM KCN, 10mM ATP, and H_2_O. Photometric measurements were then performed using a wavelength of 600nm. Finally, enzymatic activity of the ETC complex IV-enzyme cytochrome c oxidase was determined by adding a mitochondrial isolate to a solution containing DTT-reduced cytochrome c and assessing the absorbance at a wavelength of 550nm.

### Protein identification by mass spectrometry using the SWATH technique

For mass spectrometry proteins of mitochondrial isolates have been precipitated by ethanol, than homogenized in ammonium bicarbonate with a FastPrep-24 device (MP Biomedicals). Protein solution was diluted to match a protein concentration of 2μg/μl. Protein samples were reduced, carbamidomethylated and digested according to the RapidACN protocol [26]. 1 μg of the resulting peptide mixtures were subjected to SWATH-MS measurements as published previously [27]. The SWATH-library was build using the NCBInr database and the Protein Pilot 4.5 software (Sciex GmbH, Darmstadt, Germany) employing a 1% false discovery rate. Functional data for protein matches was attained using the UniProt DB.

### Transmission electron microscopy

Frozen myocardial samples were thawed and fixed in Karnovsky-fixative, then dehydrated in graded ethanols, and afterwards embedded in epoxy resin (EmBed812). 80 nm sections were double contrasted with uranyl and lead salts, and examined using the EFTEM LEO912AB (Zeiss/Oberkochen) electron microscope operating at 100 kV acceleration in zero loss mode. Images were acquired with a 1kx1k pixel side-entry mounted CCD camera controlled with the iTEM software (OSIS/Muenster), and both qualitatively and semi-quantitatively analyzed.

### Statistical analysis

Data are expressed as mean±S.E.M. or mean±SD. Differences between two analyzed groups were assessed by the Student`s t-test or ANOVA, when appropriate. Statistical significance was defined as P<0.05. SPSS Statistics (version 22, IBM, Armonk, USA) was used for data analysis.

## Results

### Hemodynamic and structural changes

Progressive pacing-induced heart failure was characterized by an enlargement of left ventricular diameters, a thinning of septal and posterior walls, and a decline of systolic function. Mean arterial blood pressure was slightly reduced in CHF, and heart rate tended to increase. Upon VPI treatment, the decline of blood pressure was more pronounced, and left ventricular dilation was moderately attenuated as compared to baseline values (ΔLVEDd in CHF-VPI vs. CHF animals 0.16±0.17 cm vs. 0.24±0.10 cm; see Table 1), even though this did not reach statistical significance.

**Table 1:** Echocardiographic and hemodynamic parameters.

	CTRL	ELVD	CHF	CHF-VPI
**IVSd [cm]**	0.30±0.03	0.25±0.03	0.25±0.02	0.24±0.04
**LVPWd [cm]**	0.27±0.02	0.27±0.04	0.23±0.03	0.24±0.03
**LVEDd [cm]**	1.33±0.14	1.65*±0.06	1.68*±0.13	1.74±0.21
**ΔLVEDd [cm]**	-	0.26±0.03	0.24±0.10	0.16±0.17
**FS [%]**	44±2	28*±1	27*±4	24*±7
**ΔFS [%]**	-	-9±4	-12±7	-13±8
**MAP[mmHg]**	73±17	71±3	64±13	51*±12
**HR [bpm]**	236±24	267±29	244±24	249±53

CTRL, control; ELVD, early left ventricular dysfunction; CHF, congestive heart failure without treatment; CHF-VPI, congestive heart failure with vasopeptidase inhibitor; IVSd, diastolic interventricular septum thickness; LVPWD, diastolic thickness of left ventricular posterior wall; LVEDd, left ventricular end-diastolic diameter; ΔLVEDd difference of left ventricular end-diastolic diameters within the same group (i.e., vs. baseline); FS, fractional shortening; ΔFS, difference of fractional shortening within the same group (i.e., vs. baseline); MAP, mean arterial pressure; HR, heart rate.

*P<0.05 vs. baseline values in each group.

### Analysis of differential protein expression patterns

#### Early left ventricular dysfunction (ELVD) vs. control (CTRL)

**Fig 2:**
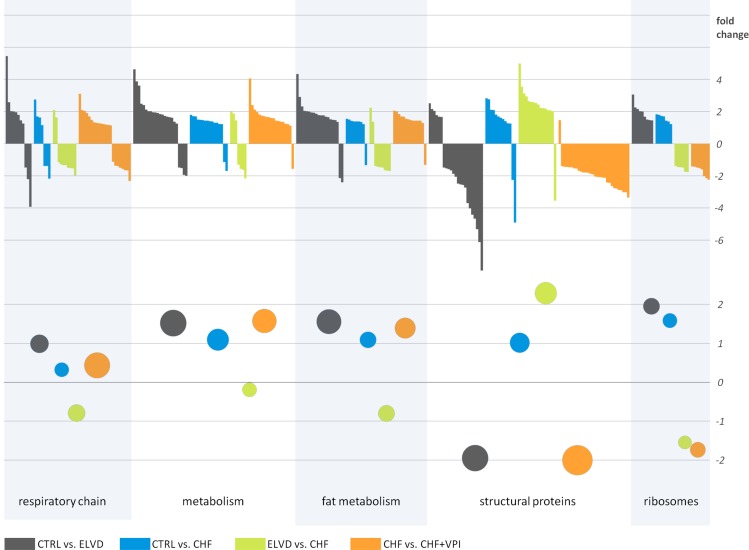
Synopsis of qualitative and quantitative proteomic results. Each color represents comparisons between two groups: gray, CTRL vs. ELVD; blue, CTRL vs. CHF; green, ELVD vs. CHF; orange, CHF vs. CHF+VPI. Data are categorized by functional assignment of the proteins to 5 classes: proteins belonging to the respiratory chain, metabolism, fat metabolism, cellular structure, and ribosomes. Each bar in the upper part of the figure represents one differently expressed protein with its fold change, respectively. Colored circles in the lower part of the figure each indicate the mean value of fold changes with the size of circles representing the number of differentially expressed proteins. The colours of the circles again reflect the comparison groups as detailed above. “Metabolism” comprises proteins with metabolic properties except for the ones belonging to “fat metabolism”.

**Fig 3:**
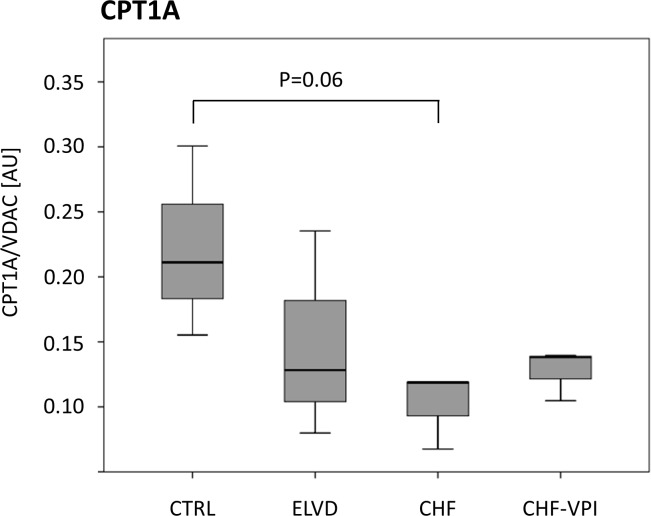
Protein expression level of carnitine palmitoyltransferase 1 A normalized to the expression of the outer mitochondrial membrane protein VDAC.

By comparing ELVD with CTRL, 199 proteins showed a significant differential expression pattern. Proteins of respiratory chain complexes I, III and V were higher expressed in ELVD. Key metabolic enzymes of glucose metabolism (i.e., glycogen phosphorylase, which catalyzes the rate limiting step of glycogenolysis, and 6-phosphofructokinase, which catalyzes the rate limiting step of glycolysis) were down-regulated in ELVD. In contrast, enzymes of the fatty acid metabolism (e.g., acetyl-CoA dehydrogenase belonging to the beta oxidative pathway) and the tricarboxylic acid (TCA) cycle were mainly up-regulated, respectively (see Fig 2). Protein expression levels of carnitine palmitoyltransferase 1 A (CPT1A), which is an essential enzyme for the beta oxidation of long chain fatty acids, tended to decrease in ELVD vs. CTRL (see Fig 3).

Finally, the majority of intermediate filament proteins were less expressed in ELVD, and ribosomal and transport proteins were mainly higher expressed (see Table 2 and Fig 2).

**Table 2:** Synopsis of different pathway regulation patterns.

Pathway	ELVD vs. CTRL	CHF vs. CTRL	CHF vs. ELVD	CHF-VPI vs. CHF
Respiratory chain complexes				
■ Complex I	↑	↑		↓
■ Complex II				↑
■ Complex III	↑	↓	↓	↑
■ Complex IV		↓		↓
■ Complex V	↑	↑	↓	↑
TCA	↑	↑	↓	
Glucose metabolism	↓	↓ / ↑	↑	~
Fatty acid metabolism	↑	↑	↓	↑
Intermediate filaments	↓	↑	↑	↓
Ribosomal proteins	↑	↑	↓	↓
Transport proteins	↑	↑		
Differentially expressed proteins (total)	199	99	106	223

### Congestive heart failure (CHF) vs. control (CTRL)

99 proteins had a significant differential expression pattern in CHF vs. CTRL. In CHF, proteins of the respiratory chain complexes I and V were up-regulated, whereas proteins of complexes III and IV were down-regulated as compared to control animals. Regarding glycolysis, one enzyme (i.e., phosphoglycerate kinase) was less expressed in CHF, and one belonging to the glycogen metabolism (i.e., glycogen debranching enzyme) showed a higher expression. Enzymes of the fatty acid metabolic pathways were up-regulated, as were proteins belonging to the TCA cycle. CPT1A expression levels were markedly reduced reaching borderline statistical significance (P = 0.058; see Fig 3). Transport proteins, ribosomal proteins, and proteins of the intermediate filaments were likewise up-regulated (see Table 2 and Fig 2).

#### Congestive heart failure (CHF) vs. early left ventricular dysfunction (ELVD)

In CHF, 106 proteins were differentially expressed as compared to ELVD. Proteins of complex III and V were uniformly down-regulated, as were enzymes of fatty acid metabolism. In contrast, glucose metabolizing proteins were higher expressed in CHF. CPT1A did not show an expression difference between CHF and ELVD (see Fig 3). TCA and ribosomal proteins were down-regulated, but proteins belonging to intermediate filaments were higher expressed in CHF (see Table 2 and Fig 2).

#### VPI-treated CHF animals (CHF-VPI) vs. untreated CHF animals (CHF)

Altogether, 223 proteins were differentially expressed in VPI-CHF as compared to CHF.

**Fig 4:**
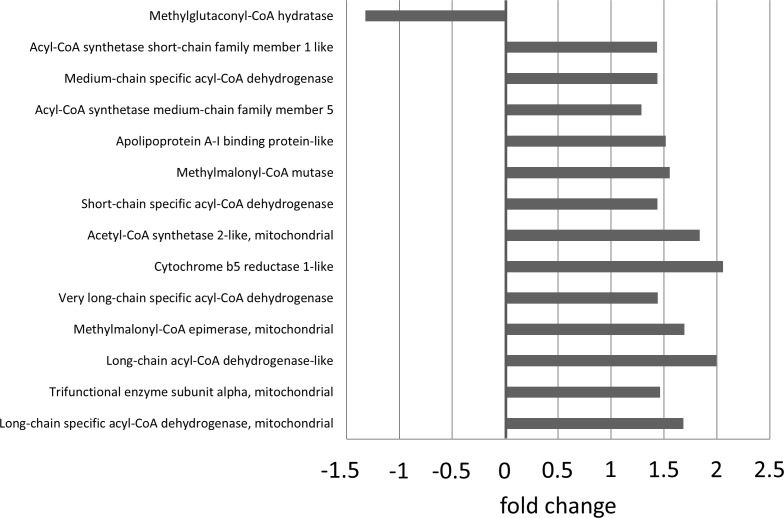
VPI-induced expression changes of enzymes belonging to the fatty acid metabolism.

Treatment with omapatrilat caused a complex change of proteins belonging to the respiratory chain complexes I-V (see Table 2 and Fig 2): whereas proteins of complex I and IV were down-regulated, proteins of complex II, III and V were up-regulated. The majority of metabolic enzymes was higher expressed in VPI-treated animals, as were proteins of fatty acid metabolism (see Fig 4), but enzymes belonging to glycolytic processes did not show distinct changes. CPT1A expression was slightly, but statistically not significant higher after VPI treatment (see Fig 3). Ribosomal proteins and proteins of intermediate filaments were down-regulated, respectively.

For further details see S1–S23 Figs of the supporting information.

### Functional analyses

#### Citrate synthase activity

**Fig 5:**
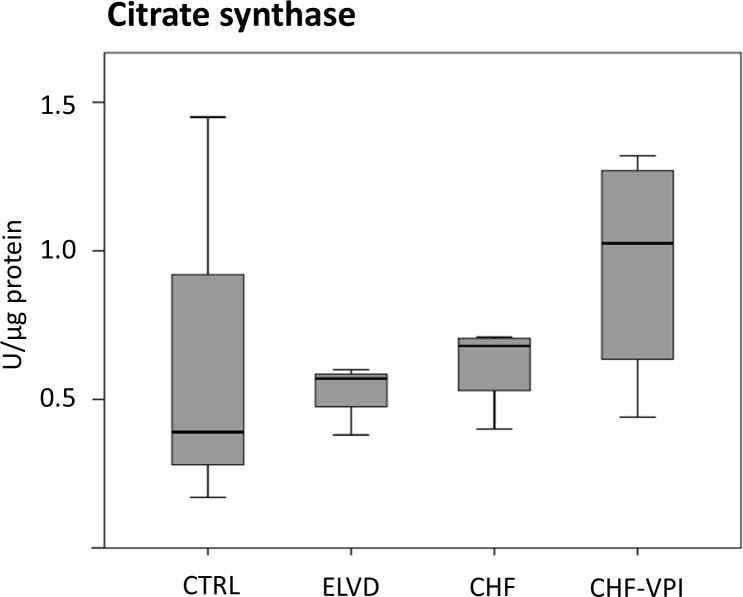
Citrate synthase activity.

To determine the functional capacity of isolated mitochondria, enzymatic activity of the key matrix marker enzyme *citrate synthase* was measured. As displayed in Fig 5, citrate synthase activity clearly tended to decrease in ELVD and CHF, but returned to near normal levels after VPI treatment.

#### Enzymatic activities of ETC complexes I, III, and IV

**Fig 6:**
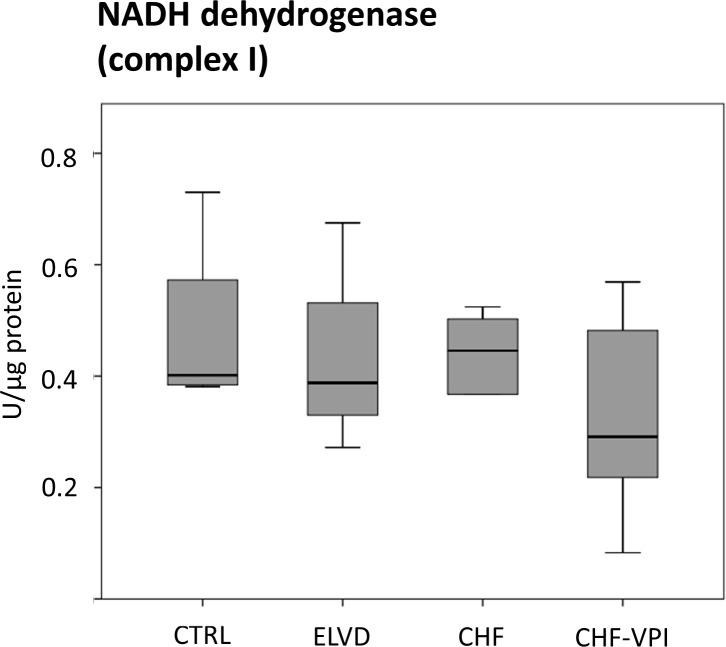
Enzymatic activity of NADH dehydrogenase (ETC complex I).

**Fig 7:**
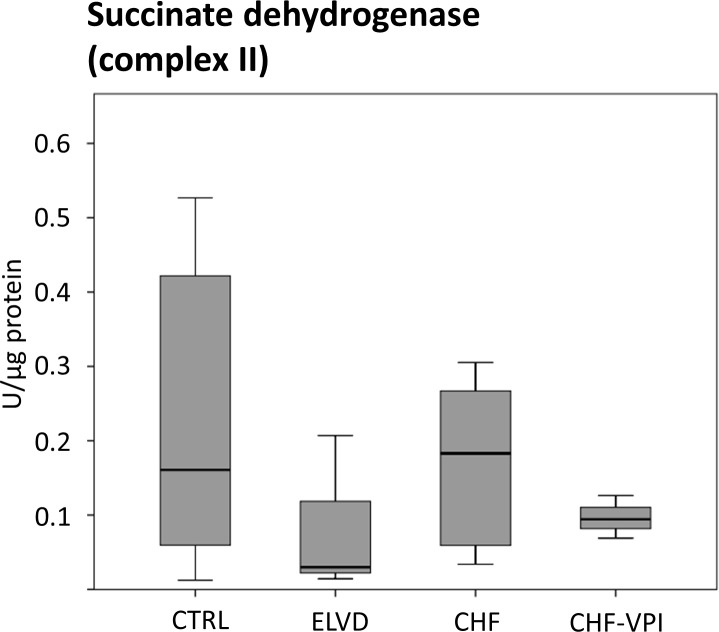
Enzymatic activity of succinate dehydrogenase (ETC complex II).

**Fig 8:**
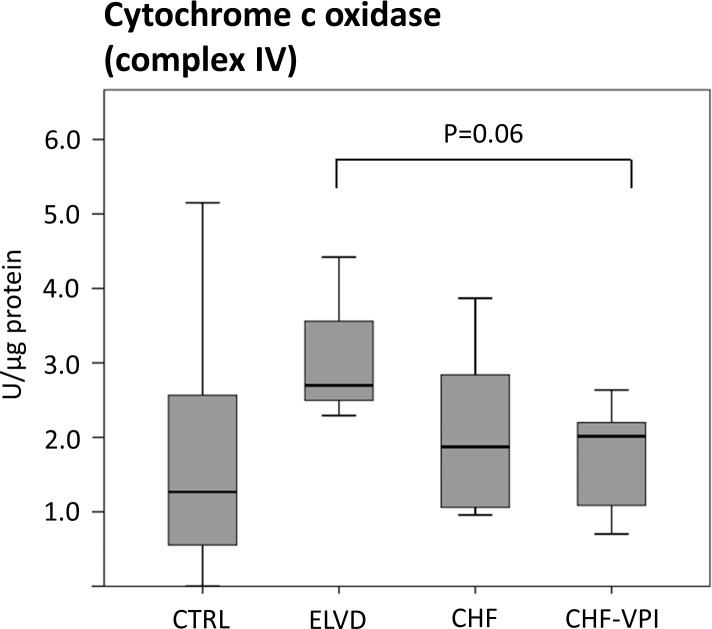
Enzymatic activity of cytochrome c oxidase (ETC complex IV).

To evaluate the functional impact of the marked and intricate expression changes of numerous ETC-proteins, we determined enzymatic activities of ETC complex I-, II-, and IV- key proteins. NADH dehydrogenase (complex I) activity was largely unchanged in ELVD and CHF as compared to CTRL, and was not relevantly influenced by VPI treatment (see Fig 6). Similarly, succinate dehydrogenase (complex II) activity did not reveal significant alterations in progressive heart failure or after VPI treatment (see Fig 7). Cytochrome c oxidase (complex IV) activity slightly increased in ELVD and decreased in CHF and CHF-VPI, reaching borderline statistical significance when comparing ELVD with CHF-VPI (P = 0.06; see Fig 8).

#### Ultrastructural analysis by transmission electron microscopy

**Fig 9:**
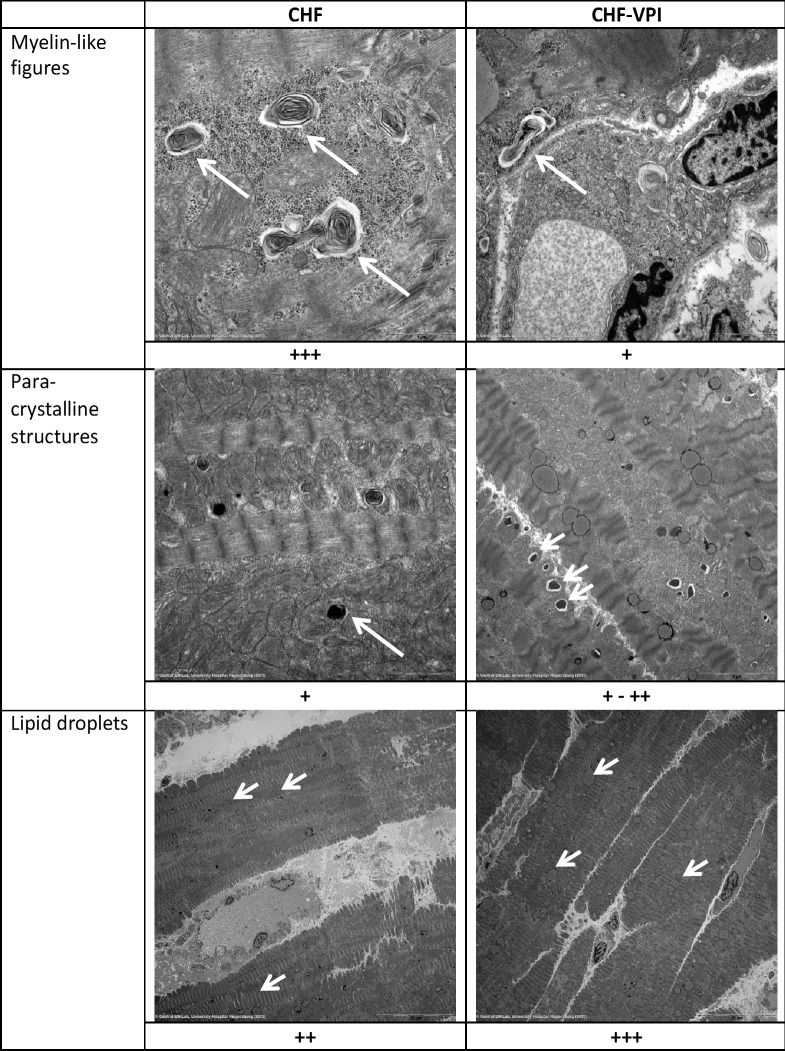
Transmission electron microscopy. By comparing CHF with CHF-VPI, differences were seen regarding myelin-like figures, paracrystalline structures, and lipid droplets. Plus signs represent the results of semiquantitative analyses.

To correlate molecular alterations with ultrastructural changes, transmission electron microscopic studies were performed in CHF and CHF-VPI animals, and differences were analyzed semiquantitatively. Three qualitative changes were found: *myelin-like structures* reflecting cellular debris digested by lysosomes were frequently seen in CHF, but seldom in CHF-VPI animals. In contrast, *paracrystalline structures* indicating degenerative processes were largely unchanged or–at best–detected slightly more often in CHF-VPI animals, than in CHF animals. Finally, *lipid inclusions* could be located slightly more frequent in CHF-VPI, than in CHF animals (see Fig 9).

## Discussion

This study evaluated molecular and ultrastructural adaptations in experimental progressive heart failure, and analyzed the effects of a combined inhibition of the renin-angiotensin system and neprilysin (RAS-/NEP-inhibition) on overt heart failure. The main findings of this work were: (1) Progressive pacing-induced heart failure is characterized by structural and functional adaptations of left ventricles, which are not substantially influenced by RAS-/NEP-inhibition, (2) progressive heart failure induces complex alterations of respiratory chain complexes, the TCA cycle and key metabolic processes, and (3) a combined inhibition of the renin-angiotensin system and neprilysin intricately influences the composition of all respiratory chain complexes, enhances the normal substrate utilizing pathway, and preserves the otherwise reduced citrate synthase activity. Furthermore, autophagolytic processes seem to be markedly reduced upon RAS-/NEP-inhibition.

### RAS-/NEP-inhibition does not substantially influence left ventricular structural remodeling

Progressive tachycardia-induced heart failure was characterized by structural and functional adaptations, which are also hallmarks of the human disease [28,29]. Interestingly, left *ventricular* remodeling was not substantially influenced by combined RAS-/NEP-inhibition in this study, whereas left *atrial* remodeling was markedly attenuated in our previous work investigating omapatrilat in the same animal model [15]. It is noteworthy that very similar findings were recently reported for the RAS-/NEP-inhibitor LCZ696 in the PARAMOUNT trial [30], which compared LCZ696 to valsartan in patients with HFPEF (i.e., heart failure with preserved ejection fraction): in this study parameters of left *atrial* remodeling were significantly improved after 36 weeks, whereas indicators for left *ventricular* remodeling remained unchanged. From this follows that a combined RAS-/NEP-inhibition seems to influence left atrial rather than left ventricular structural remodeling, whereas profound molecular alterations are also evident in left ventricles.

### Progressive heart failure is characterized by profound alterations of the mitochondrial proteome

Components of the respiratory chain complexes were very dynamically altered during progression to overt heart failure. This finding extends the results of our previous proteomic analyses, which were conducted in unfractionated left ventricular tissue samples [17] by unveiling a rather complex regulation pattern: Enzymes belonging to complexes I and V, which are the starting and end points of respiration, were higher expressed in early left ventricular dysfunction (ELVD) and overt heart failure (CHF) as compared to control animals, respectively. Contrarily, proteins of complex V were down-regulated in CHF vs. ELVD, which means that the highest expression levels were found in ELVD. Finally, proteins of complex III were up-regulated in ELVD and down-regulated in CHF, as were proteins belonging to complex IV. Despite these intricate protein expression changes, enzymatic activities of ETC complexes I, II, and IV were largely unaltered during progression to overt heart failure, which is in good accordance with the results of one previous study using the same model of heart failure [31]. Interestingly, enzymatic activities of the remaining two ETC complexes III [32,33] and V [33,32], which were not evaluated in our study,seem to be *reduced* in pacing-induced heart failure. Thus, protein expression levels do not obligatorily reflect enzymatic activity, which is not unusual and can be due to several reasons (e.g., enzymatic inactivation by posttranslational modifications [34]), but might be of particular importance in mitochondria: Increasing evidence points to a fundamental role of supramolecular aggregates (i.e., “respirasomes”) for effective oxidative phosphorylation [35]. Since respirasomes are assembled by a specific combination of respiratory chain complexes, any compositional change of individual complexes might hamper their organization to supramolecular aggregates and therefore impair proper respiratory functionality. This is supported by Rosca and coworkers, who found a dramatic decrease of ADP-stimulated respiration in a canine model of coronary microembolization-induced heart failure, which was caused rather by a defective respirasome assembly than by an impaired enzymatic activity of individual respiratory chain complexes [35]. From this follows, that protein expression changes do not necessarily imply that enzymatic activities are likewise altered, and, furthermore, unchanged enzymatic activities do not necessarily reflect a proper mitochondrial respiration. Given these highly complex relationships, it would be mandatory to assess both structural (i.e., composition of the individual ETC complexes *and* aggregation of ETC complexes to respirasomes) and functional (i.e. enzymatic activities *and* mitochondrial respiration) properties of the ETC in order to comprehensively evaluate its functionality. Beyond respiratory chain complexes, two other crucial mitochondrial pathways were found to be profoundly altered in our study: substrate utilization (i.e., beta oxidation and glycolysis) and the Krebs cycle (i.e., TCA cycle). Enzymes of glycolysis and, partly, glycogenolysis showed lower expression patterns by comparing ELVD with control animals and CHF with control animals, respectively, but were higher expressed in CHF as compared to ELVD. This means that glucose metabolizing processes remain down-regulated throughout progression to overt heart failure, even though this down-regulation is somehow attenuated by proceeding from ELVD to CHF. Contrarily, enzymes of fatty acid degrading beta-oxidation were up-regulated by comparing ELVD with control, and CHF with control animals, respectively, but were down-regulated during transition from ELVD to CHF. These findings generally confirm a `metabolic switch`in heart failure, which is characterized by a down-regulation of beta-oxidation and an up-regulation of glycolysis [36], but add an important fact: Obviously, the principal metabolic set-up of the healthy heart with a predominance of beta-oxidation over glycolysis persists or even increases throughout progression to heart failure, and is just contrarily modulated as soon as LV dysfunction reaches its terminal stage. Similarly, components of the TCA cycle were up-regulated in ELVD and CHF as compared to control animals, but were down-regulated in CHF vs. ELVD indicating a likewise attenuation of this persistently up-regulated pathway in later stages of the disease. Interestingly, enzymatic activity of citrate synthase, which catalyzes the first step of the Krebs cycle in a pace-making fashion, was reduced in both ELVD and CHF. Against this background it would be tempting to speculate that the initial up-regulation of TCA cycle enzymes would have to compensate for the reduced substrate flow through the enzymatic starting point of this pathway representing a mechanism which is exhausted with further progression to overt heart failure. This could be supported by Dodd and coworkers, who investigated Krebs cycle activity in a model of myocardial-infarction induced heart failure by demonstrating a functional TCA cycle impairment, which began to correlate with the degree of cardiac dysfunction not until 6 weeks after induction of myocardial infarction [37]. Thus, in the early stages of heart failure compensatory mechanisms could probably counterbalance the reduced citrate synthase activity, which was also seen in the study of Dodd et al. [37], but this could be exhausted in later stages.

### Combined RAS-/NEP-inhibition has profound impact on mitochondrial key pathways

It is noteworthy that the highest number of differentially expressed proteins was found by comparing the CHF-VPI with the CHF group, which underlines the far-reaching impact of RAS-/NEP-inhibition on the mitochondrial molecular setup. In detail, as compared to CHF animals, RAS-/NEP-inhibition did affect three central mitochondrial pathways (i.e., electron transfer chain (ETC), substrate utilization, and TCA cycle), and severely influenced autophagolytic processes. Regarding ETC, treatment with omapatrilat reduced the expression of proteins belonging to complexes I and IV, and increased components of complexes II, III, and V. This partly reversed the adaptations of complexes I and III (which are main sources of reactive oxygen species in mitochondria [38]) in progressive heart failure and aggravated those of complexes IV and V. Interestingly, a deficiency of complex II and III has been linked to human dilated cardiomyopathy [39] and ischemic cardiomyopathy [40], respectively, so a restoration of complex II and III content might probably counteract LV dysfunction. Besides RAS-/NEP-inhibition, also TNF-alpha blockade by etanercept [32], cardiac resynchronization by biventricular pacing [41], treatment with resveratrol in doxorubicin-induced heart failure [42], or application of trimetazidine [43] were likewise followed by ETC adaptations, thereby indicating a therapeutic involvement of the ATP-generating apparatus in these instances. But given the intricate processes around oxidative phosphorylation, which include the complex functionality of supramolecular aggregates (as mentioned above), the true functional relevance of these findings (e.g., reduction of oxidative stress or augmented efficiency of ATP generation) is difficult to delineate and has clearly to be evaluated in focused future experiments.

Beyond ETC adaptations, RAS-/NEP-inhibition increased the expression of proteins involved in the metabolism of free fatty acids (FFA), but did not relevant influence glycolytic pathways in our study. This might counteract the relative down-regulation of beta-oxidation in progressive heart failure and suggests some restoration of the normal substrate utilization processes. Interestingly, a similar effect has been shown for failing human hearts which have been unloaded by left ventricular assist devices [44] or for recovering canine hearts after halting the tachypacing stimulus [45], so this metabolic change might indicate some kind of cardiac regeneration by RAS-/NEP-inhibition. Similarly, activity of the key TCA enzyme citrate synthase, which was reduced in both ELVD and CHF, clearly tended to normal levels in omapatrilat-treated animals, even though protein expression of TCA components *per se* was not altered. A likewise reconstitution of enzymatic activity was again seen in unloaded human [44] and recovering canine hearts [45], so this omapatrilat-induced change might in turn mirror some extent of molecular reverse remodeling.

Finally, we could demonstrate a remarkable reduction of myelin-like figures along with an increase of paracrystalline structures in cardiomyocytes of omapatrilat-treated animals, whereas lipid droplets, which were already highly expressed in CHF animals, did show only marginal changes. Myelin-like figures, which were also found in cardiomyopathic hamsters in addition to signs of autophagic cell death [46] or in cyclophosphamide-damaged cardiomyocytes [47], are supposed to be rather *late* products of autophagic lysosomal digestion [48]. In contrast, lipid droplets seem to *rapidly* evolve in damaged skeletal muscle cells [48] and are indicative for a disturbance of lipid metabolism leading to lipid accumulation in the heart [49]. Lastly, paracrystalline structures have been linked to mitochondrial diseases [50] and could be a sign of degeneration, though their significance might not easily be interpreted in our study. Taken together, these omapatrilat-induced ultrastructural adaptations most probably point to a beneficial decrease of otherwise detrimental autophagic processes, which usually accompany progressive left ventricular dysfunction.

### Limitations

Our work might have some limitations: Firstly, even though we conducted our experiments by using a cutting edge proteomic approach with some additional functional tests, this was nevertheless a classical proteomic study, so we cannot clearly specify the functional relevance of our findings. Otherwise, this was actually beyond the scope of our work, which was rather conceived to depict a global view of molecular alterations in properly isolated cardiac mitochondria thereby providing a starting point for subsequent functional analyses. Secondly, the combined RAS-/NEP-inhibition by omapatrilat significantly lowered blood pressure, which is a well known effect of this compound. Consequently, one might ascribe the beneficial action of omapatrilat to this hemodynamic property rather than to an intrinsic modulatory effect on neurohumoral activation. That being said very recent evidence with the closely related substance *LCZ696*, which blocks neprilysin and the angiotensin receptor instead of the angiotensin converting enzyme, rather argue for an intrinsic neurohumoral modulatory action as a cause of the beneficial drug effects [51]. Facing these hemodynamic side effects of a combined RAS-/NEP-inhibition, but also considering the current standard of care (which is represented by an ACE inhibitor much more than by a placebo preparation), an active comparator such as enalapril could be advantageous. Thirdly, we used the vasopeptidase inhibitor (VPI) omapatrilat instead of the newer angiotensin receptor neprilysin inhibitor (ARNI) *LCZ696* to execute a combined RAS-/NEP-inhibition, since the latter one was not available at the beginning of our experiments. Given the closely related modes of action, one could argue for a class effect, which means that our results should be transferable. But this has clearly to be confirmed as soon as LCZ696 becomes available for research purposes.

### Conclusion

Progressive pacing-induced left ventricular dysfunction is characterized by profound constitutional alterations of the electron transfer chain, the TCA cycle, and substrate utilization processes. These adaptations are at least partly reversed by RAS-/NEP-inhibition, which also seems to reduce detrimental subcellular degeneration in cardiomyocytes. Thus, RAS-/NEP-inhibition unfolds beneficial effects on energetically relevant pathways in a model of progressive pacing-induced heart failure.

## Supporting Information

S1 Fig Mitochondrial ETC proteins in CTRL vs. ELVD. (TIF)

S2 Fig Metabolic enzymes in CTRL vs. ELVD. (TIF)

S3 Fig Intermediate filaments in CTRL vs. ELVD. (TIF)

S4 Fig Fat metabolism in CTRL vs. ELVD. (TIF)

S5 Fig Ribosomal proteins in CTRL vs. ELVD. (TIF)

S6 Fig Transport proteins in CTRL vs. ELVD. (TIF)

S7 Fig Mitochondrial ETC proteins in CTRL vs.CHF. (TIF)

S8 Fig Metabolic enzymes in CTRL vs. CHF. (TIF)

S9 Fig Intermediate filaments in CTRL vs. CHF. (TIF)

S10 Fig Fat metabolism in CTRL vs. CHF. (TIF)

S11 Fig Ribosomal proteins in CTRL vs. CHF. (TIF)

S12 Fig Transport proteins in CTRL vs. CHF. (TIF)

S13 Fig Mitochondrial ETC proteins in CHF vs. CHF+VPI. (TIF)

S14 Fig Metabolic enzymes in CHF vs. CHF+VPI. (TIF)

S15 Fig Intermediate filaments in CHF vs. CHF+VPI. (TIF)

S16 Fig Ribosomal proteins in CHF vs. CHF+VPI. (TIF)

S17 Fig Transport proteins in CHF vs. CHF+VPI. (TIF)

S18 Fig Mitochondrial ETC proteins in ELVD vs. CHF. (TIF)

S19 Fig Metabolic enzymes in ELVD vs. CHF. (TIF)

S20 Fig Intermediate filaments in ELVD vs. CHF. (TIF)

S21 Fig Fat metabolism in ELVD vs. CHF. (TIF)

S22 Fig Ribosomal proteins in ELVD vs. CHF. (TIF)

S23 Fig Transport proteins in ELVD vs. CHF. (TIF)
