# Supplementary figures and images for: Combined Inhibition of the Renin-Angiotensin System and Neprilysin Positively Influences Complex Mitochondrial Adaptations in Progressive Experimental Heart Failure
Source: PLoS One. 2017 Jan 11;12(1):e0169743. doi: 10.1371/journal.pone.0169743 (PMC5226780; doi:10.1371/journal.pone.0169743)

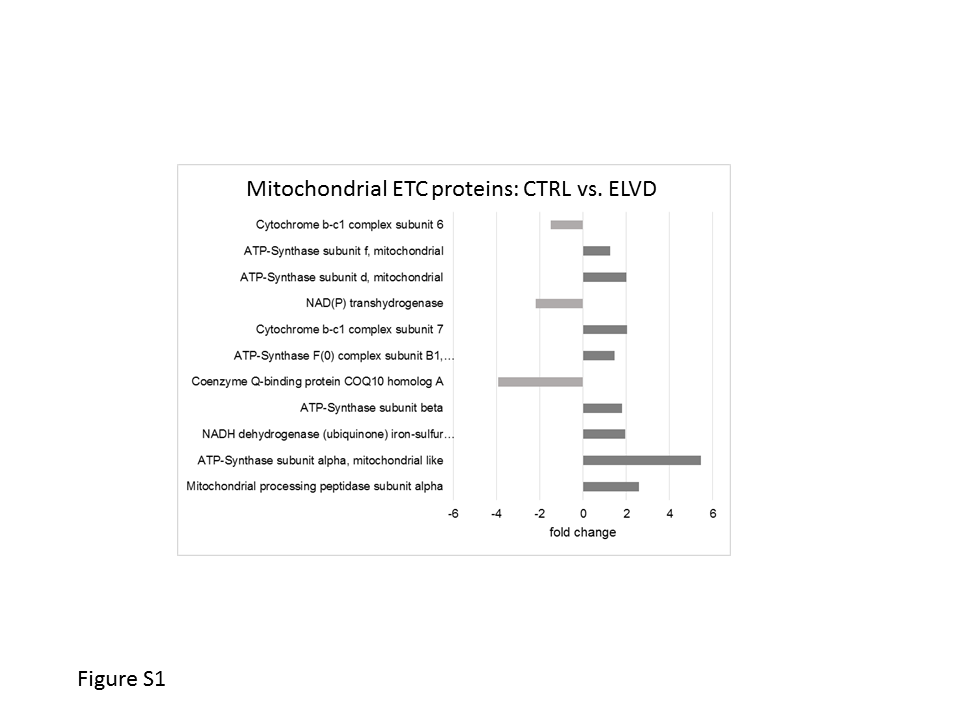

Supplement: S1 Fig — (TIF) [file pone.0169743.s001.tif]

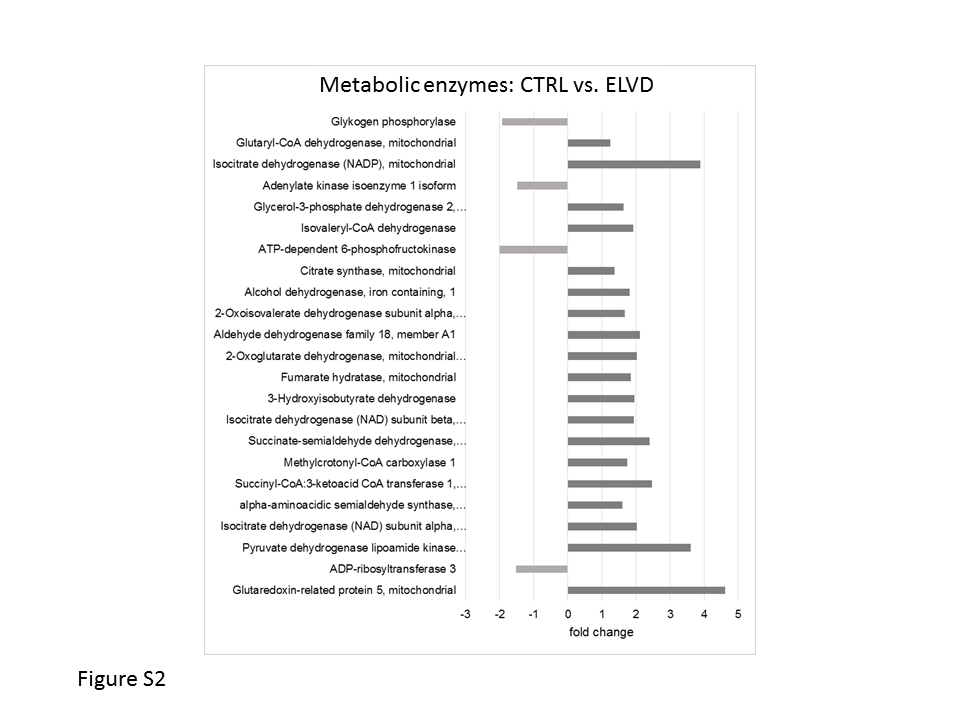

Supplement: S2 Fig — (TIF) [file pone.0169743.s002.tif]

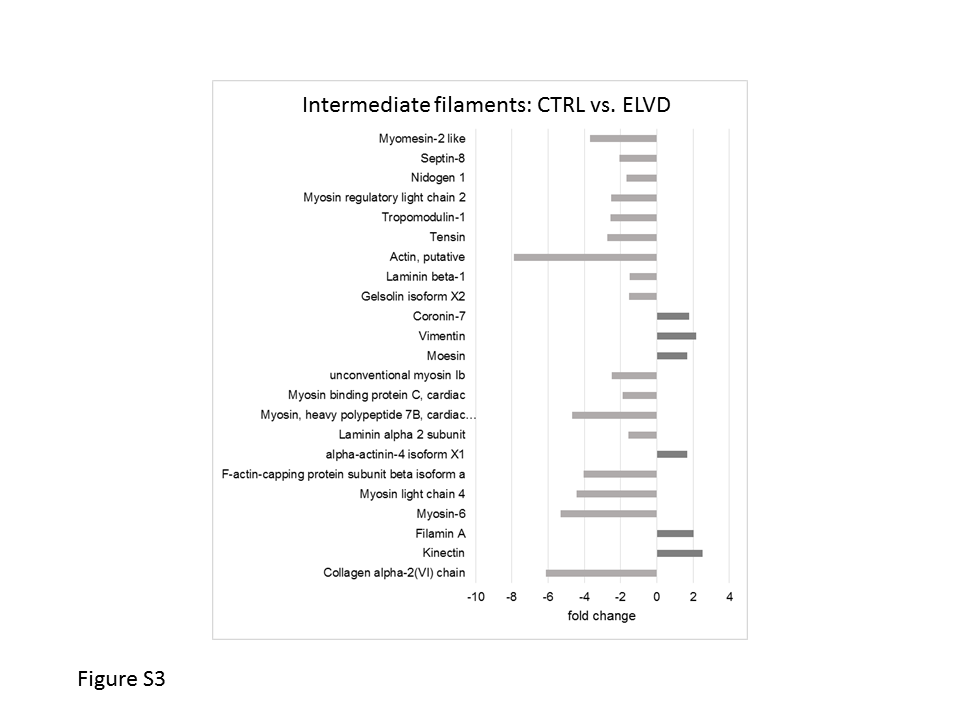

Supplement: S3 Fig — (TIF) [file pone.0169743.s003.tif]

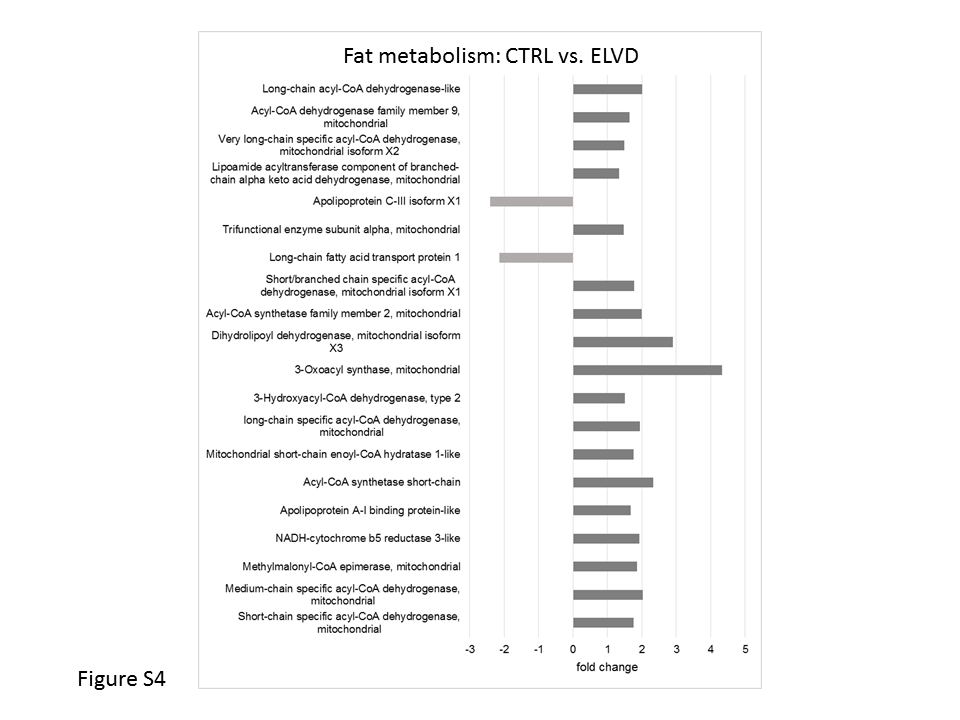

Supplement: S4 Fig — (TIF) [file pone.0169743.s004.tif]

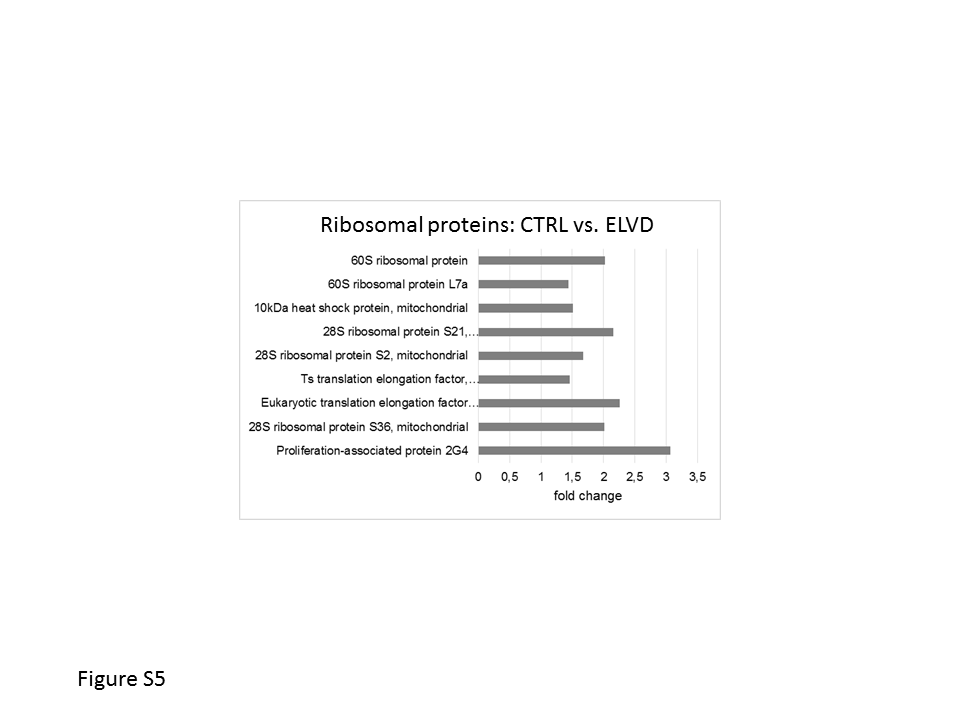

Supplement: S5 Fig — (TIF) [file pone.0169743.s005.tif]

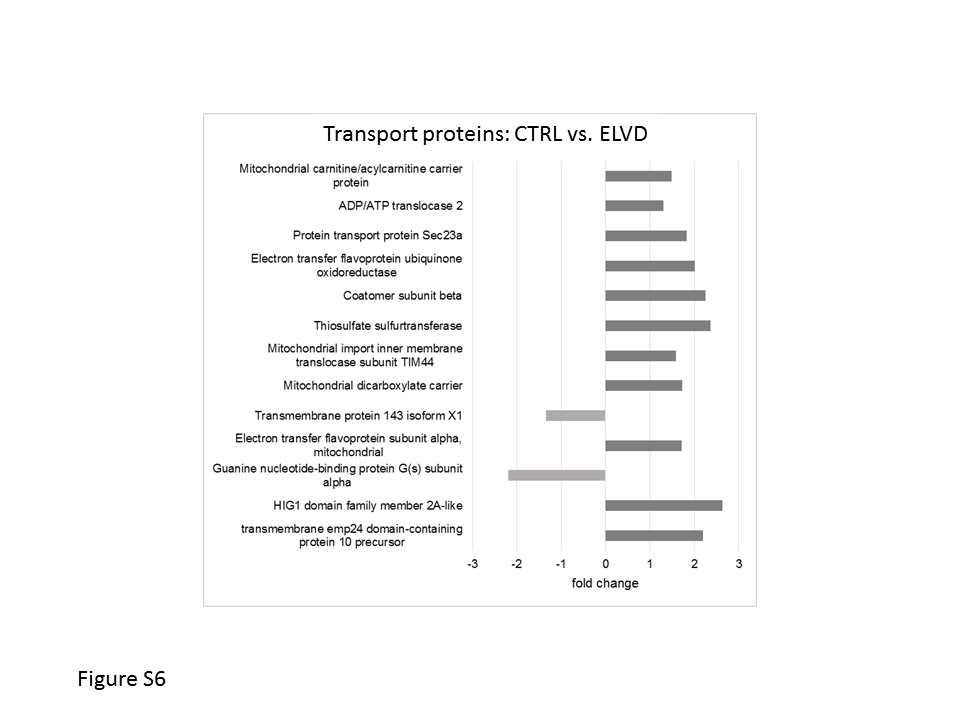

Supplement: S6 Fig — (TIF) [file pone.0169743.s006.tif]

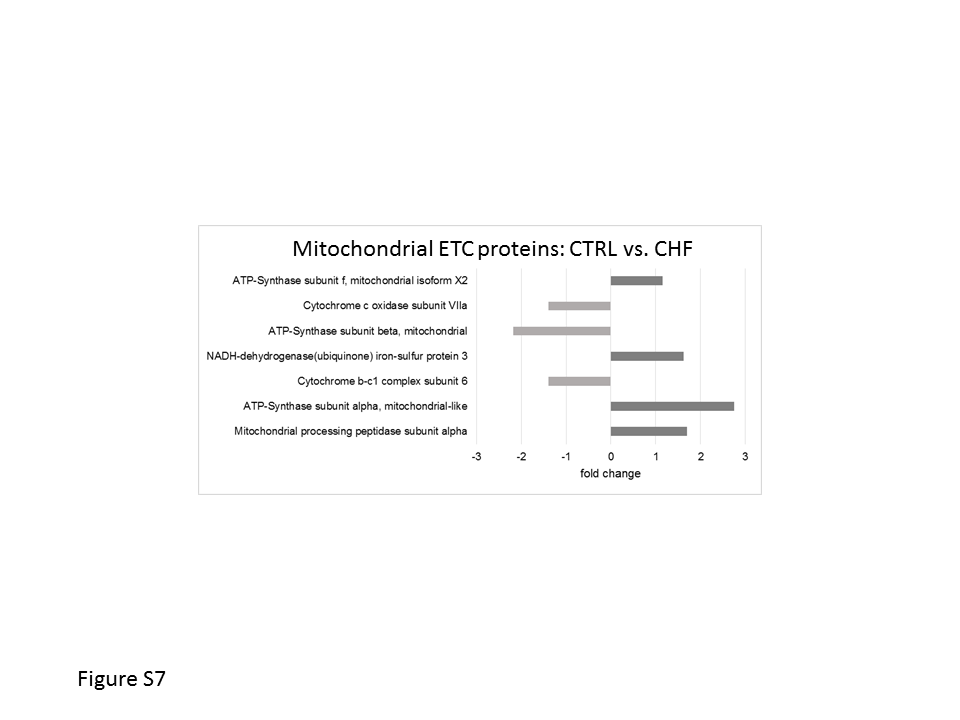

Supplement: S7 Fig — (TIF) [file pone.0169743.s007.tif]

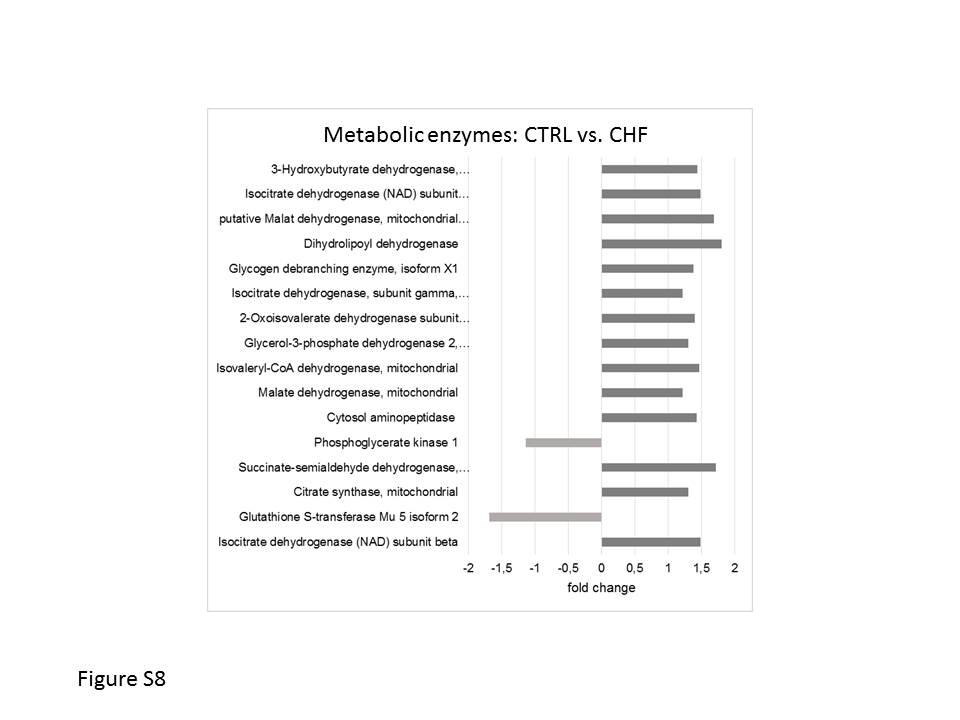

Supplement: S8 Fig — (TIF) [file pone.0169743.s008.tif]

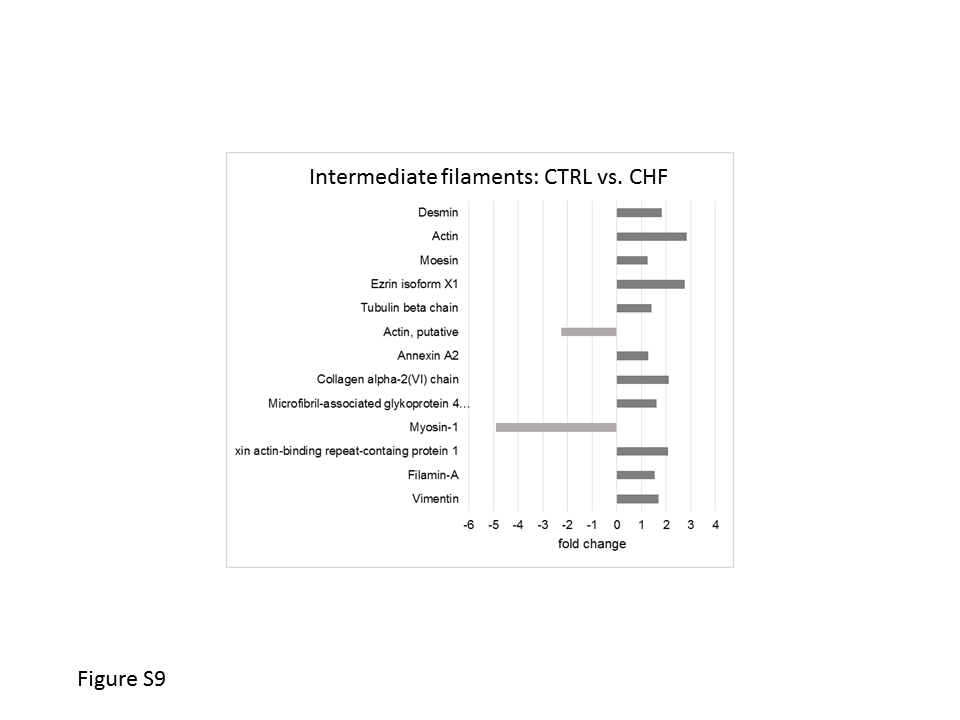

Supplement: S9 Fig — (TIF) [file pone.0169743.s009.tif]

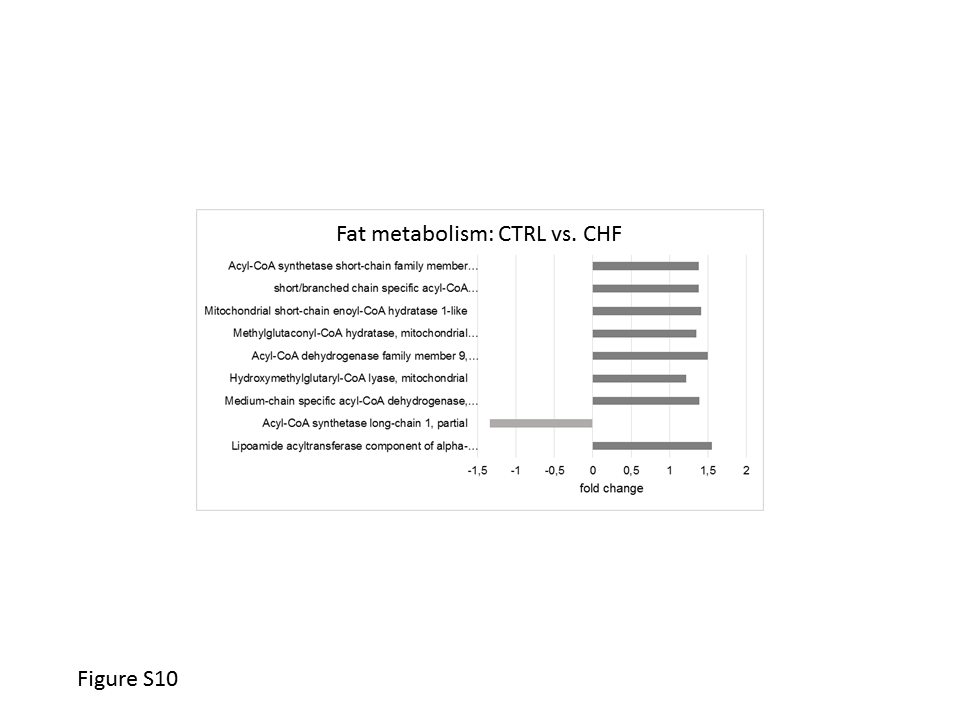

Supplement: S10 Fig — (TIF) [file pone.0169743.s010.tif]

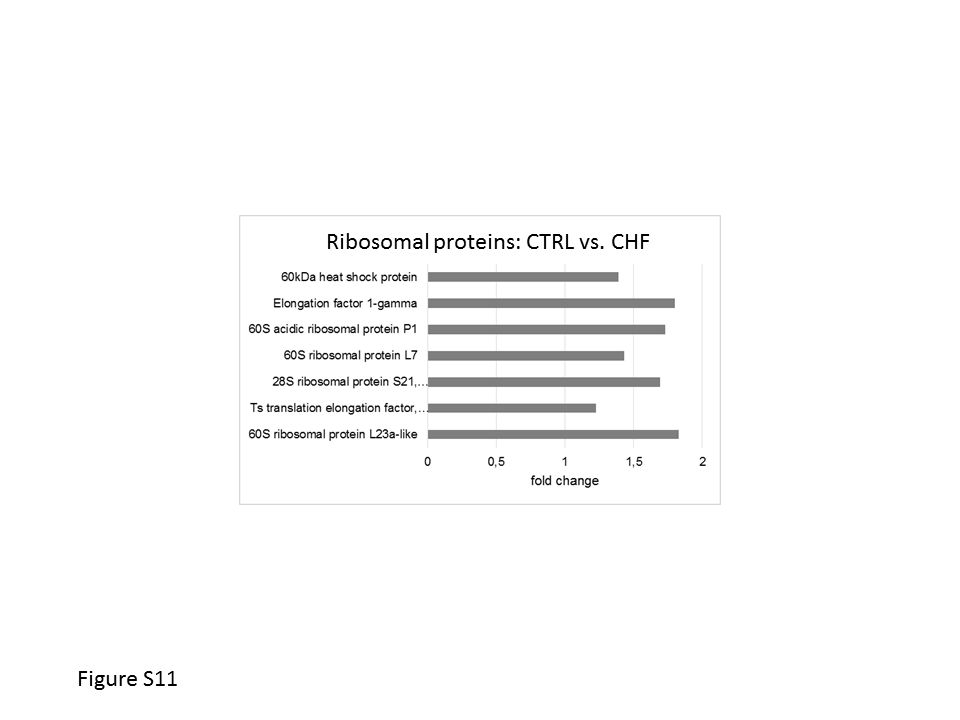

Supplement: S11 Fig — (TIF) [file pone.0169743.s011.tif]

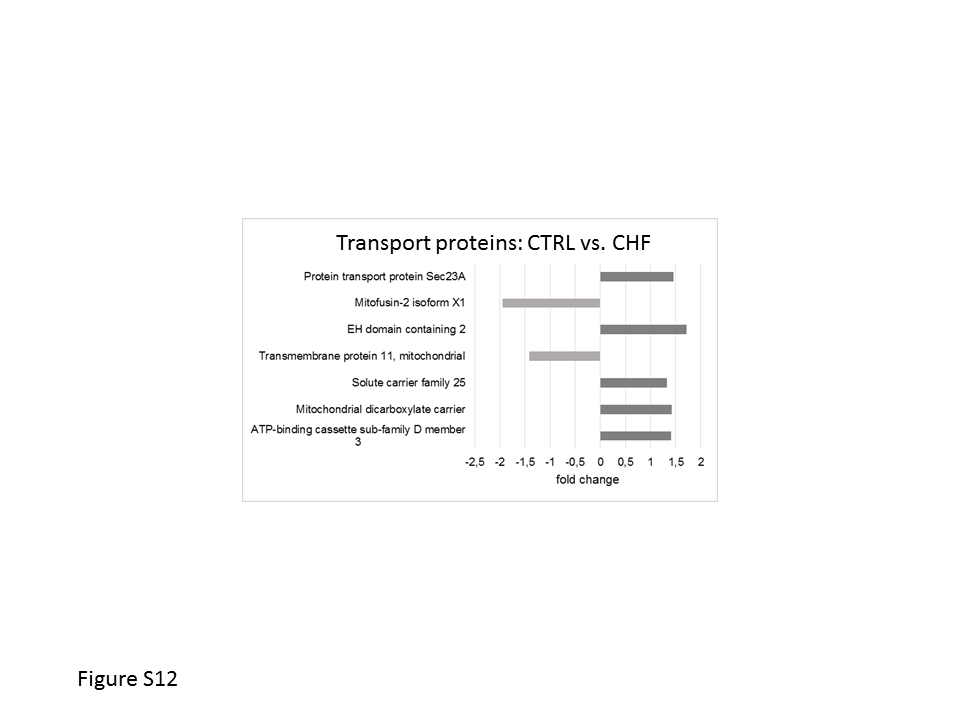

Supplement: S12 Fig — (TIF) [file pone.0169743.s012.tif]

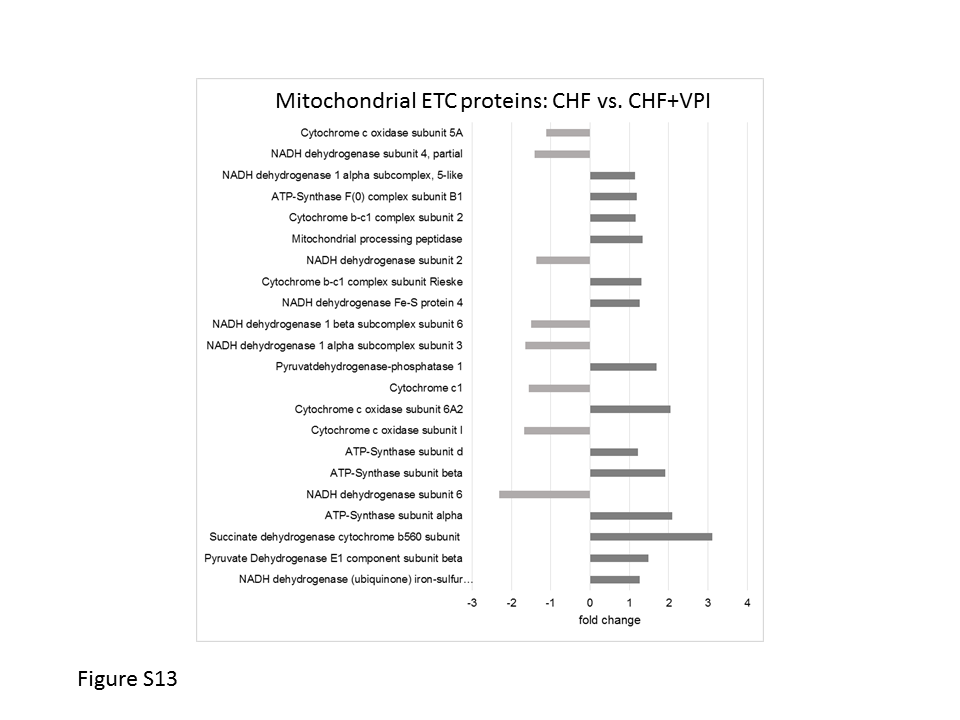

Supplement: S13 Fig — (TIF) [file pone.0169743.s013.tif]

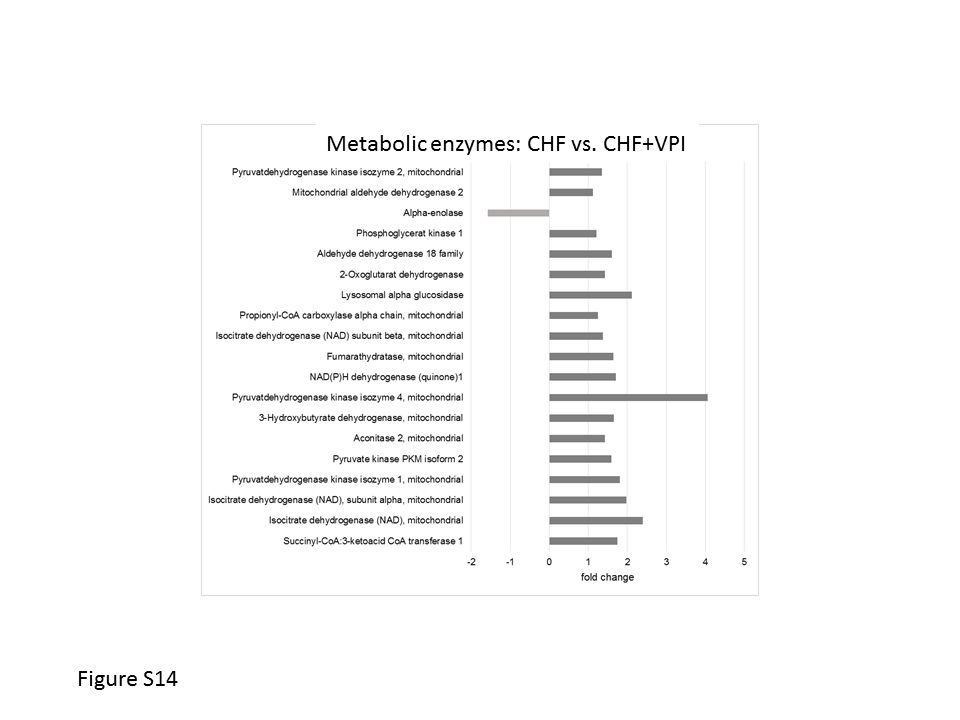

Supplement: S14 Fig — (TIF) [file pone.0169743.s014.tif]

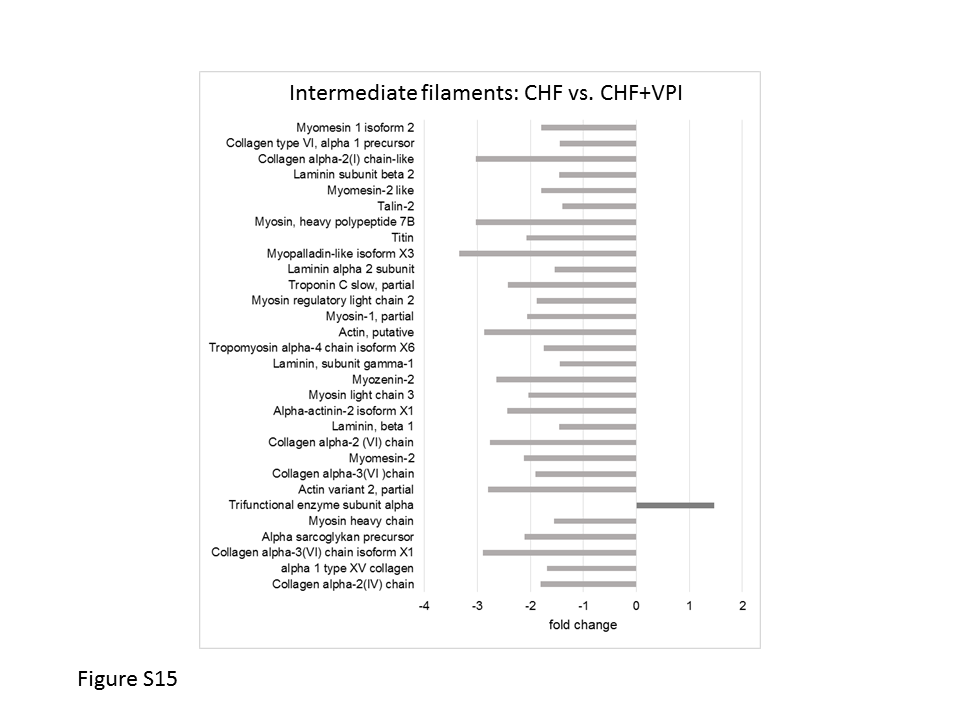

Supplement: S15 Fig — (TIF) [file pone.0169743.s015.tif]

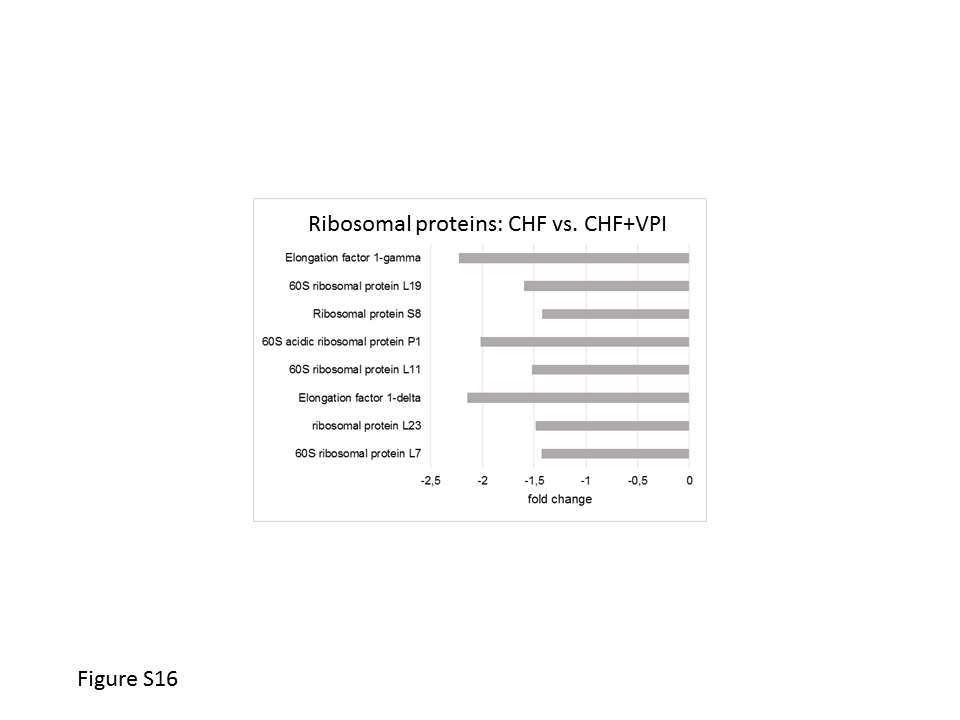

Supplement: S16 Fig — (TIF) [file pone.0169743.s016.tif]

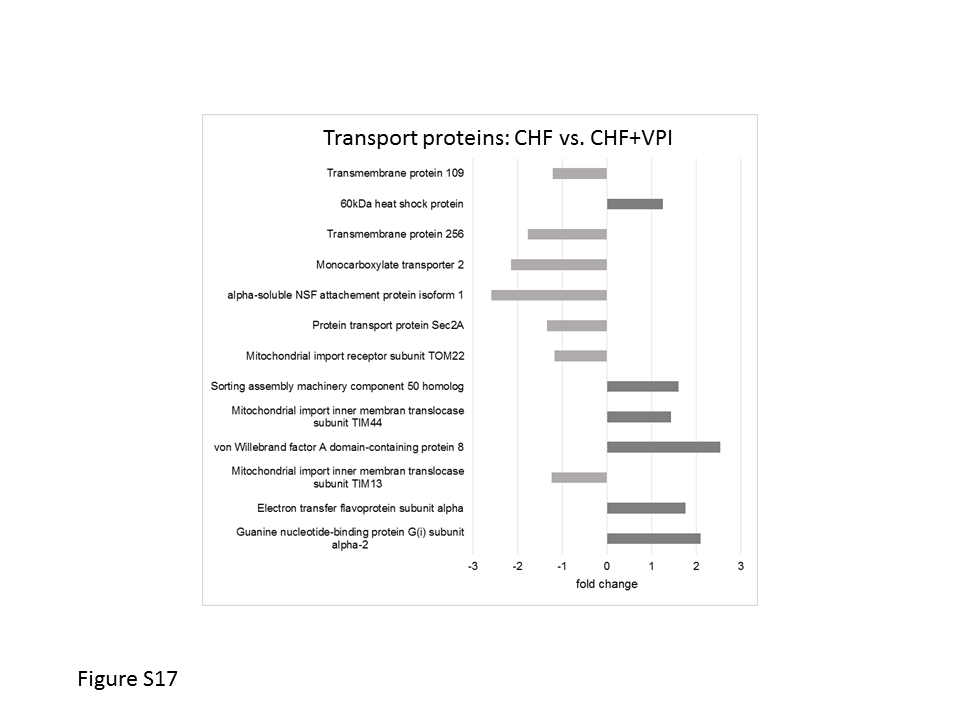

Supplement: S17 Fig — (TIF) [file pone.0169743.s017.tif]

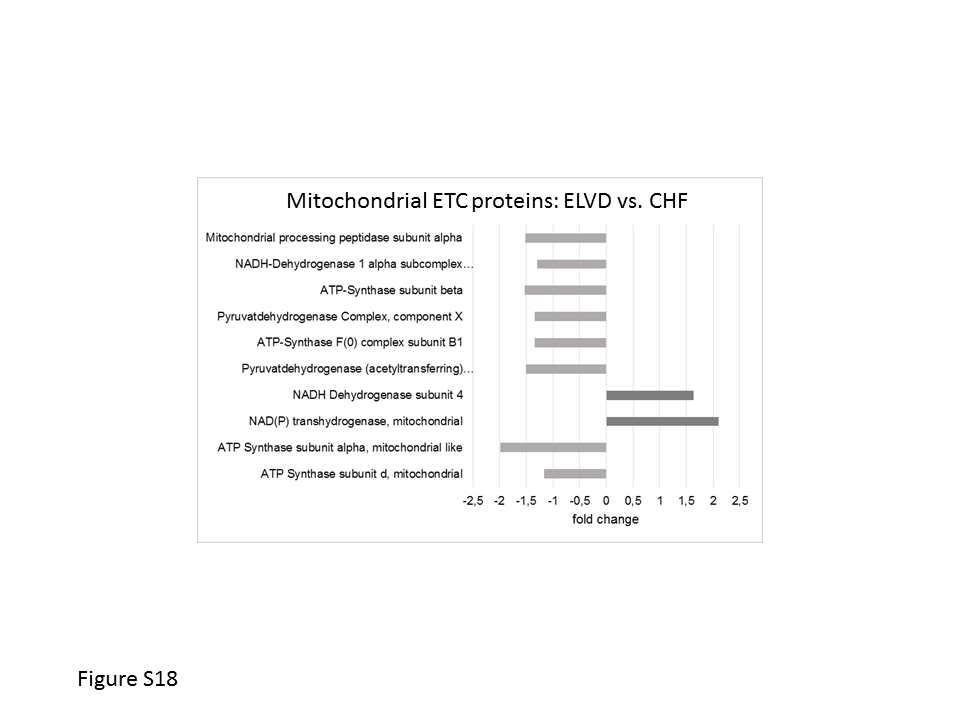

Supplement: S18 Fig — (TIF) [file pone.0169743.s018.tif]

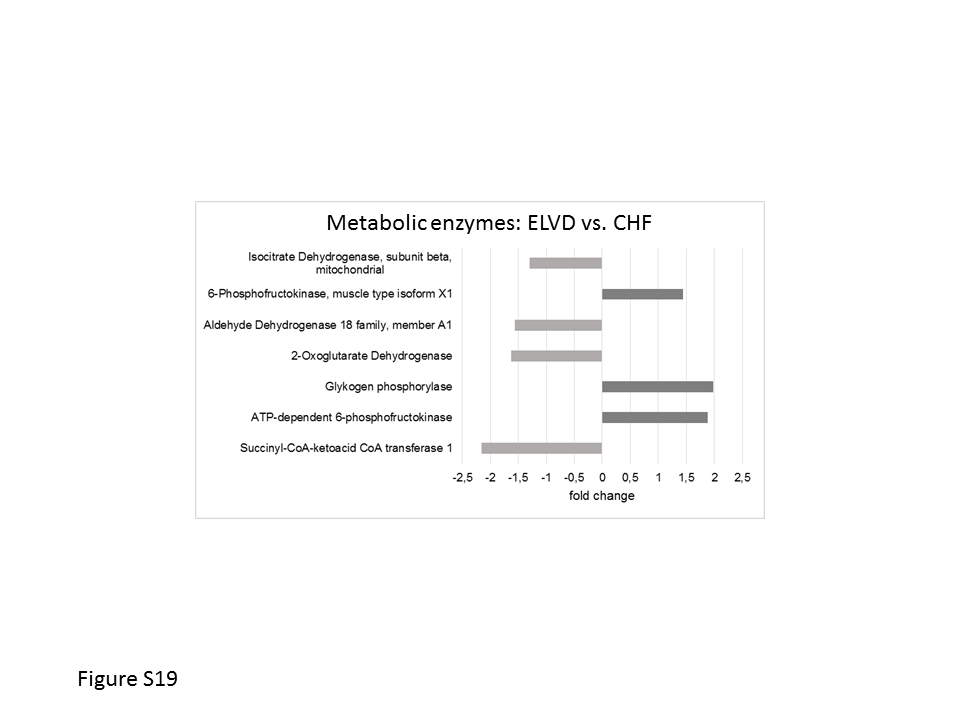

Supplement: S19 Fig — (TIF) [file pone.0169743.s019.tif]

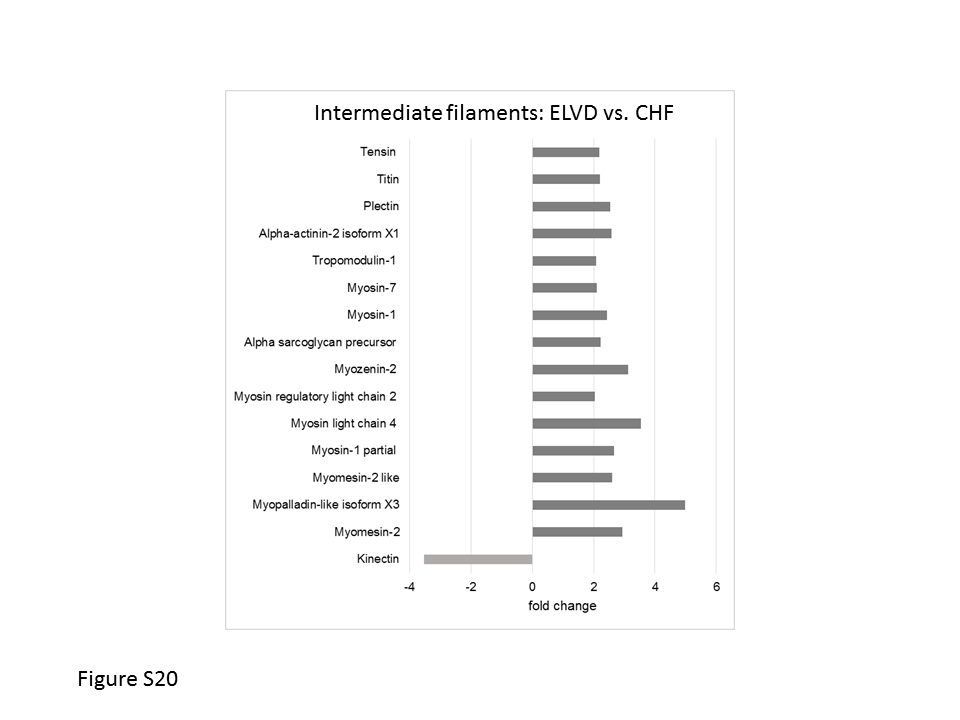

Supplement: S20 Fig — (TIF) [file pone.0169743.s020.tif]

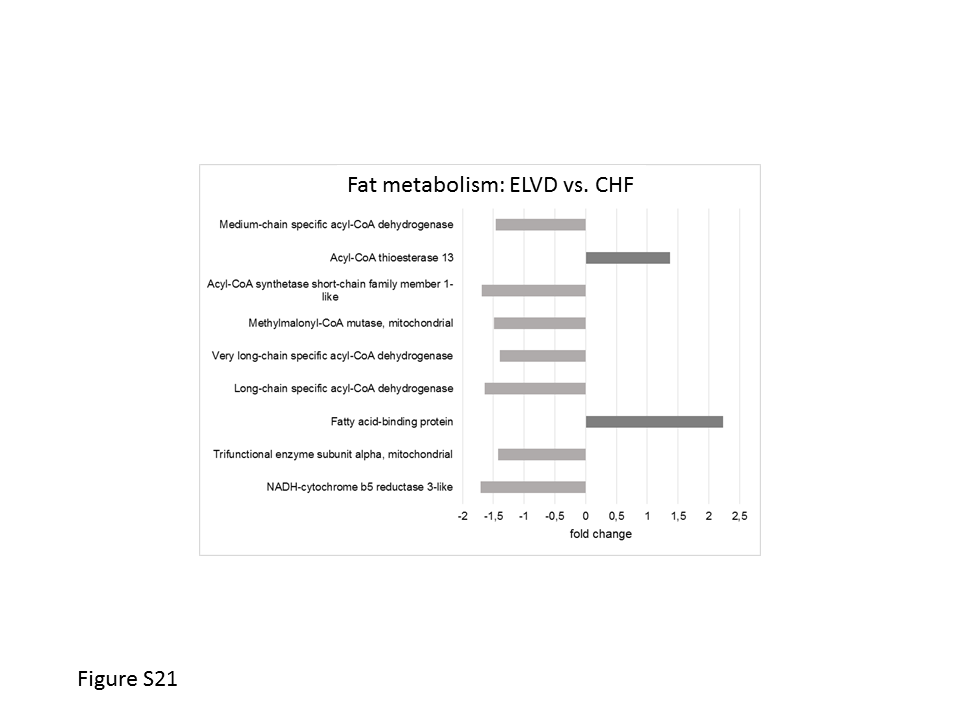

Supplement: S21 Fig — (TIF) [file pone.0169743.s021.tif]

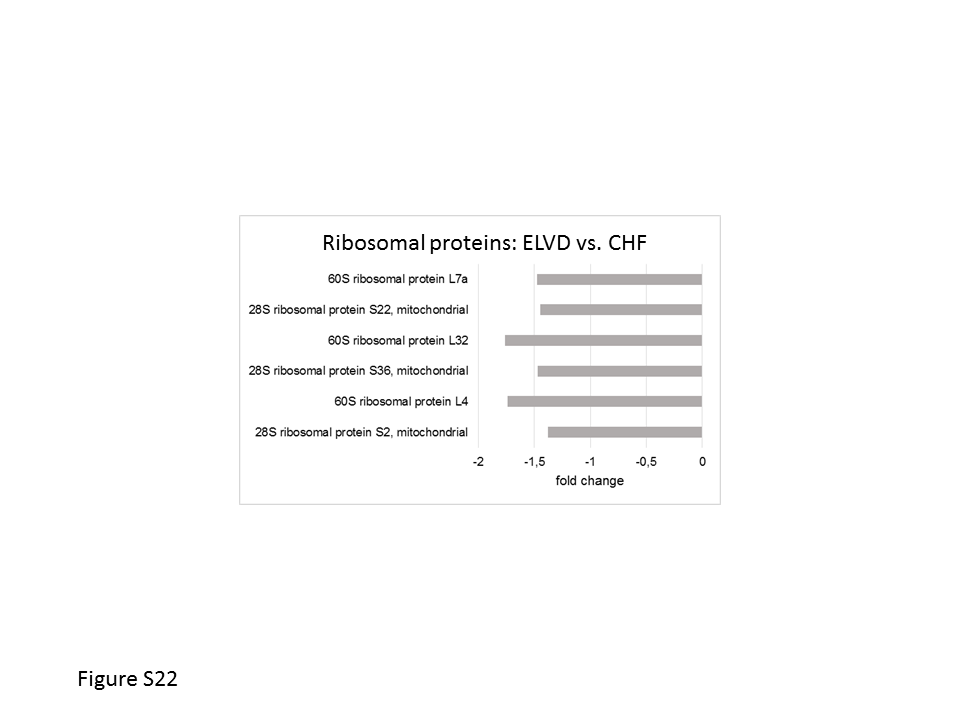

Supplement: S22 Fig — (TIF) [file pone.0169743.s022.tif]

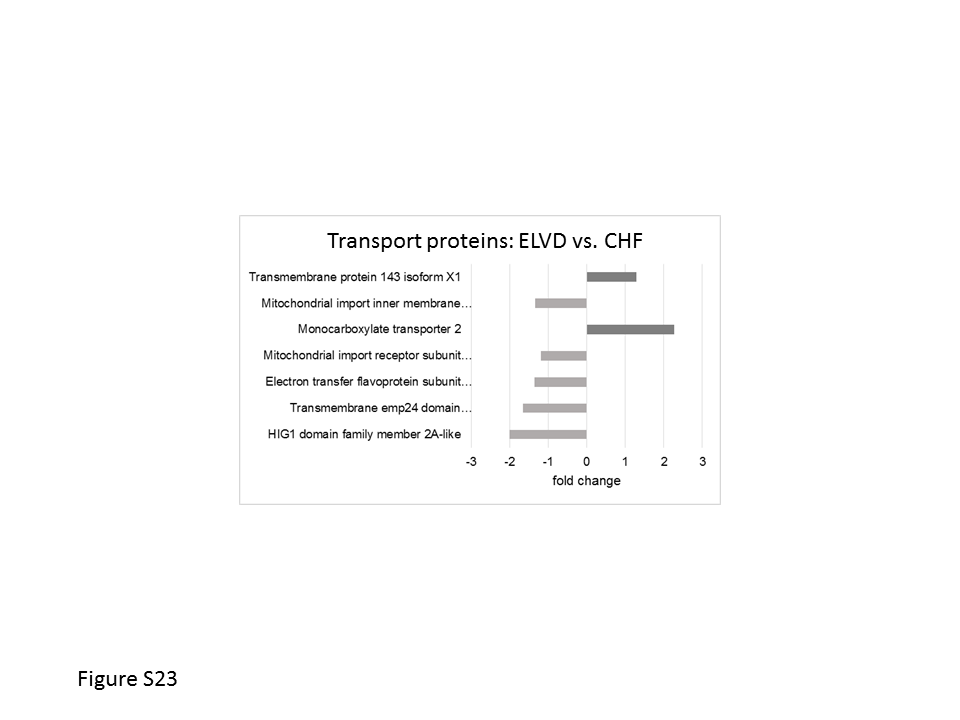

Supplement: S23 Fig — (TIF) [file pone.0169743.s023.tif]
